# Defining high-risk pregnancy: Protocol for a systematic scoping review of clinical determinants, complications, and adverse birth outcomes

**DOI:** 10.1371/journal.pone.0334326

**Published:** 2025-10-15

**Authors:** Xiali Yang, Xin Wee Chen, Philip R. A. Baker

**Affiliations:** 1 School of Nursing, Ningxia Medical University, Ningxia, China; 2 Department of Public Health Medicine, Faculty of Medicine, Universiti Teknologi MARA, Sungai Buloh, Selangor, Malaysia; 3 Faculty of Health, School of Public Health and Social Work, Queensland University of Technology, Brisbane, Queensland, Australia; 4 Graduate School of Public Health, Yonsei University, Seoul, South Korea; 5 Australian Centre for Health Law Research, Queensland University of Technology, Brisbane, Queensland, Australia; University of Campinas, BRAZIL

## Abstract

**Introduction:**

Accurately identifying clinical determinants within the medical paradigm is essential for evidence-based planning and management of high-risk pregnancies, and for preventing adverse birth outcomes through targeted interventions. However, a systematic synthesis of regional classification systems, risk factors, complications, and adverse birth outcomes associated with high-risk pregnancies remains lacking.

**Objective:**

This study protocol aims to systematically identify relevant published research, assess the risks of bias, and summarize the routinely collected clinical determinants/data used in classifying high-risk pregnancy status and the relationship of this status with its complications.

**Methods and analysis:**

The bibliographic databases of Cochrane Library, Web of Science, CNKI, SCOPUS, and PubMed-MEDLINE will be searched for observational studies without language restrictions. The two-stage screening process will be conducted, involving independent full-text reviews, risk of bias assessments, and data extraction by two reviewers. Narrative synthesis will address selective publication bias using established critical appraisal and evidence evaluation methods. Study selection and reporting will adhere to the PRISMA-P guidelines.

**Ethics and dissemination:**

Ethics approval is not required, as this systematic review utilises only published data. The findings will be disseminated through publication in a peer-reviewed journal and presentation at conferences.

**Protocol registration:**

PROSPERO database registration (registration number: CRD420251026327).

## Introduction

High-risk pregnancy refers to a clinical classification for pregnant women at an elevated risk of complications or adverse outcomes affecting mothers and/or neonates. While broadly defined as pregnancies with higher risks compared to the general population [1], there is no universal consensus on its definition, which varies across medical guidelines, organizations, and regions. The lack of standardization complicates the interpretation of routinely collected antenatal data used to classify high-risk pregnancies. Adverse birth outcomes, such as maternal mortality and postpartum hemorrhage, are critical global public health challenges, contributing significantly to child morbidity and mortality by impacting short- and long-term health outcomes [2]. Risk factors for high-risk pregnancies can be grouped into five categories: demographic factors, adverse pregnancy history, medical complications, pregnancy-related conditions, and environmental or social determinants [3]. Common outcomes include preterm birth, low birth weight, and small-for-gestational-age (SGA) infants, which are major contributors to neonatal mortality and long-term health issues [4].

Globally, 27% of births are small for gestational age (SGA) [5], 1.3% are stillbirths [6], 10.6% are preterm [7], and 2.2% involve structural congenital anomalies [8]. High-risk pregnancies consistently demonstrate higher probabilities of adverse outcomes compared to low-risk cases [9–12]. Adverse outcomes arise from multifactorial etiologies, including genetic, biological, behavioral, environmental, and social factors [4]. Cross-sectional studies have identified context-specific sociodemographic disparities, lower socio-economic status, suboptimal pre-pregnancy BMI, pre-pregnancy, substandard housing conditions, previous obstetric complications, maternal comorbidities, clinical conditions, nutritional status, and underutilization of prenatal care associated with adverse birth outcomes [13–19]. Socioeconomic predictors are well-known, but actionable clinical factors during routine antenatal care are underexplored. Addressing these gaps is crucial to meet the ENAP 2030 goals of reducing neonatal mortality and stillbirths to ≤12 per 1,000 births [20].

The clinical markers routinely collected during antenatal care to identify high-risk pregnancies are poorly defined. Clarifying these determinants is essential to understanding high-risk pregnancies, reducing adverse birth outcomes, and improving targeted interventions. However, a comprehensive systematic review linking clinical determinants, complications, and maternal and neonatal outcomes is lacking. This review aims to address these gaps by identifying key determinants and their associations with outcomes.

### Review questions

1. Which socio-economic determinants and routinely collected clinical determinants are used to classify a high-risk pregnancy?
2. What is the relationship between the high-risk pregnancy status and its complications?

## Methods

### Study design

This protocol follows PRISMA-P guidelines (PRISMA-P) [21] and the PRISMA statement, including the flow chart, to track search records. It is registered with PROSPERO [registration number: CRD420251026327].

### Search strategy

The Cochrane Library, Web of Science, CNKI, SCOPUS, and PubMed (MEDLINE) will be searched for observational studies from 2014 onwards. Studies must describe risk-factor classification systems, the risk factors defining them, and their association with adverse birth outcomes. The search strategy will follow the POE framework: Population/Problem (high-risk pregnancy classification), Outcome (pregnancy complications and adverse outcomes), and Exposure (risk factors Search strategies will use Mesh-indexed keywords combined with Boolean operators (AND/OR), including terms like “risk factor,” “high-risk pregnancy,” “pregnancy complication,” and “adverse birth outcome.” The draft search strings for all databases are provided in S1 File. Data management will involve Excel and EndNote software to identify and remove duplicates electronically and manually.

All non-English studies meeting eligibility criteria will be manually translated by bilingual team members, with subsequent grammar and clarity checks performed using Grammarly (Premium version) to ensure linguistic accuracy. No automated translation tools will be used without human verification. The search will be constructed using keywords and limiters appropriate to the database:

- (high-risk pregnan* OR at-risk pregnan* OR high risk prenan*) AND
- (risk factor* OR predictor* OR determinant*) AND

(pregnan* complication OR adverse pregnan* outcome OR adverse obstetric outcome OR birth outcome OR maternal outcome)

### Study selection

The review will investigate and explore the factors proposed to define high-risk pregnancies, with a focus on the information routinely collected during antenatal appointments. These include maternal age, BMI, low education, low household income, historical obstetric and gynaecological complications, surgical history, and fetal biometry identified through ultrasound, such as fetal heart, femur length, abdominal circumference, and biparietal diameter.

**Fig 1:**
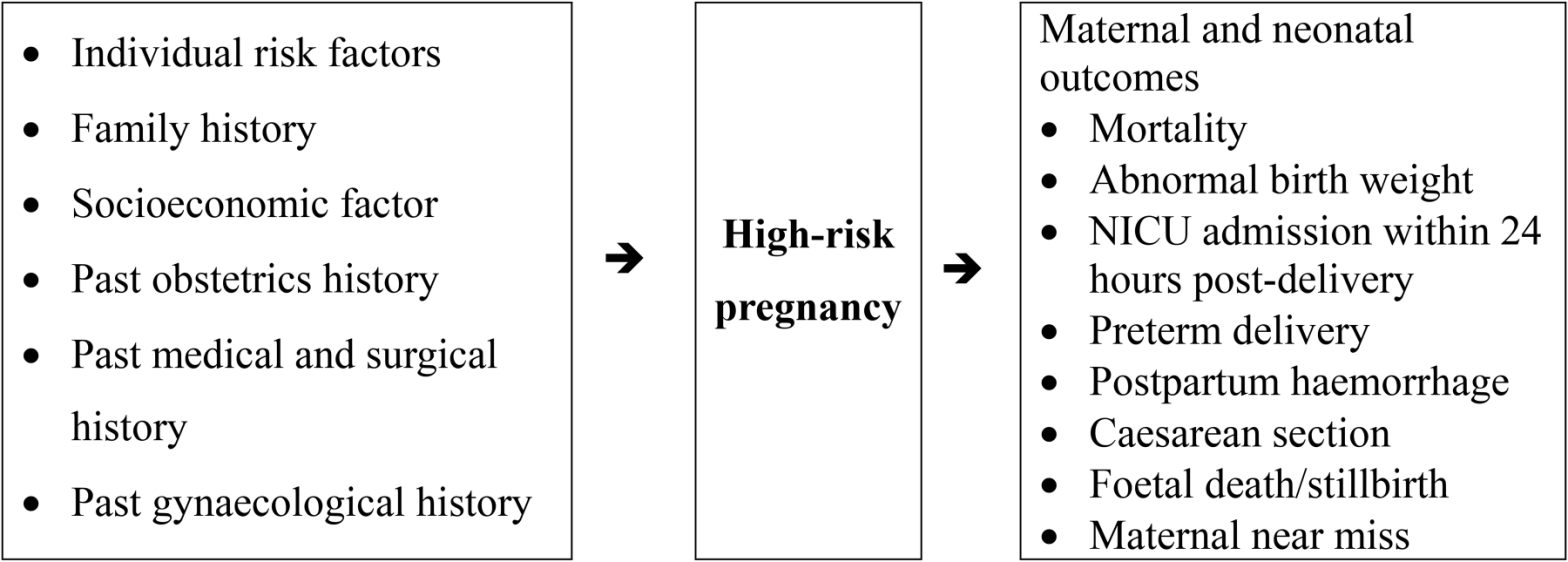
Logic model and conceptual framework (Descriptions of variables are detailed in Table 1).

Adverse birth outcomes will encompass pregnancy, labour, and delivery complications affecting newborn health, ranging from mild to severe and with potential long-term impacts (Fig 1 and Table 1). The review will focus on prevalent maternal and neonatal outcomes:

**Table 1:** Descriptions of routinely collected clinical determinants/data.

Variable name	Descriptions
Individual Risk Factors	Age, parity, education level, height (short stature)
Family History	Hypertension, heart disease, diabetes
Socioeconomic factor	Number of antenatal checkups, presence of long-distance marriage, household income level, healthcare distance from home
Past Obstetrics History	History of abortion, preterm birth, cesarean section
Past medical and surgical history	Comorbidity (Tuberculosis, heart disease, diabetes mellitus, etc., history of other special, major surgical procedures, and history of drug allergy
Past gynecological history	Genital tract deformity, uterine fibroids or ovarian cysts, history of vaginal and cervical surgery (e.g., intrauterine/ laparoscopic surgery)

(i) maternal and neonatal mortality (defined as death status),
(ii) abnormal birth weight (macrosomia defined as birth weight >4 kg and low birth weight defined as <2500g), and
(iii) neonatal intensive care unit (NICU) admission within 24 hours post-delivery
(iv) preterm delivery (<37 weeks)
(v) postpartum hemorrhage (defined as bleeding volume >500ml after vaginal delivery or >1000ml after cesarean section)
(vi) Cesarean section
(vii) Fetal death/stillbirth (defined as intrauterine death occurring at ≥20 weeks of gestation, acknowledging that the specific gestational age threshold may vary across included studies)
(viii) Maternal near miss (defined according to the WHO criteria as a woman who nearly died but survived a complication that occurred during pregnancy, childbirth, or within 42 days of termination of pregnancy)

The review will include observational studies (cross-sectional, case-control, and cohort studies) from 2014 to 2024 that recruit mothers with high-risk pregnancy status and contain information on maternal and neonatal outcomes. Eligible studies must report identified determinants in pregnancy (exposures of high-risk pregnancy) and conclude with maternal and neonatal outcomes (pregnancy complications and adverse outcomes). We will exclude studies with no maternal or neonatal outcomes reported at the delivery stage, and those with unavailable full-text manuscripts despite attempts to contact the corresponding authors. Protocols, ongoing studies, and studies without ethical approval will be listed but not included. The language of publication will be inclusive through the use of translation applications.

The literature search and record screening have not yet started. Record screening is expected to begin in August 2025, followed by data extraction in October 2025. Results synthesis and reporting are expected to be finished by 1 December 2025.

### Data extraction and analysis

A two-stage screening process will be carried out using EndNote software: the initial stage will involve title and abstract screening by a single reviewer (YX), while the subsequent stage will include full-text screening by two independent reviewers. Discrepancies will be resolved by a third reviewer. Data extraction will adhere to a structured protocol, with YX conducting the data extraction and CXW verifying its accuracy; PB will settle disputes. A pre-tested Microsoft Excel spreadsheet will be used to extract predefined details: authors, study settings (e.g., country, location, and duration), respondent numbers, study design, risk factors, pregnancy complications, and adverse outcomes. Primary outcomes will comprise delivery complications and adverse birth outcomes, along with the identified high-risk pregnancy determinants.

### Methodological quality assessment

Two reviewers will independently evaluate study quality using the Newcastle-Ottawa scale for non-randomised studies [22–23]. A third reviewer will resolve any discrepancies in the assessments.

### Data synthesis

Descriptive synthesis will summarise the included studies’ population characteristics, context, methods used, and the results reported, including any statistical descriptors in a tabular format. All eligible studies will be reported. Data will be interpreted in the context of the identified risks of bias, selective reporting of outcomes, and the precision of the findings. To strengthen the narrative synthesis, we will thematically categorise risk-factor classification systems using the PEO framework. Finally, we will develop a comprehensive conceptual framework to visualise risk factor-outcome relationships across studies.

## Discussion

Improving pregnancy outcomes, managing high-risk pregnancies, and reducing maternal and neonatal mortality are central goals of strengthening maternal and child health initiatives globally [24–26]. This review aims to identify classification systems for high-risk pregnancies and map them to maternal and neonatal outcomes. Understanding the distribution and interplay of high-risk factors is crucial for guiding health management priorities and informing policy decisions. This review seeks to identify the strengths and limitations of the existing evidence, focusing on determinants of adverse birth outcomes. However, psychosocial factors (e.g., inter-partner violence, postnatal depression) are beyond the scope of this review.

High-risk pregnancy is a complex condition requiring early detection, precise risk assessment, and holistic management to optimize maternal and neonatal outcomes [27–28]. A fuller understanding of classification could leverage machine learning and AI approaches to incorporate additional variables, enabling dynamic risk assessments and improving antenatal detection of high-risk pregnancies.

Limitations of this review will be the ability to explore in detail the psychosocial domain (e.g., inter-partner violence, post-natal depression as an outcome) [29].

## Supporting information

S1 File Search strategy. (DOCX)
