# Supplementary material for: Defining high-risk pregnancy: Protocol for a systematic scoping review of clinical determinants, complications, and adverse birth outcomes
Source: PLoS One. 2025 Oct 15;20(10):e0334326. doi: 10.1371/journal.pone.0334326 (PMC12527127; doi:10.1371/journal.pone.0334326)
Supplement: S1 File — (DOCX) [file pone.0334326.s001.docx]

**Supporting Information 1: Search strategy**

**Cochrane Library**

#1 (MeSH descriptor: [High-Risk Pregnancy] explode all trees) OR (high-risk pregnan*:ti,ab,kw)

#2 (MeSH descriptor: [Risk Factors] explode all trees) OR (MeSH descriptor: [Pregnancy Complications] explode all trees) OR (pregnancy complication*:ti,ab,kw)

#3 (MeSH descriptor: [Infant, Low Birth Weight] explode all trees) OR (MeSH descriptor: [Premature Birth] explode all trees) OR (adverse birth outin come*:ti,ab,kw)

#4 #1 AND #2 AND #3

#5 Publication Year from 2014 to 2024

**Web of Science**

#1 TS=("high risk pregnan*" OR "high-risk pregnan*" OR "pregnancy risk factor*")

#2 TS=("pregnancy complication*" OR "obstetric complication*" OR "risk factor*")

#3 TS=("adverse birth outcome*" OR "preterm birth" OR "stillbirth" OR "low birth weight")

#4 #1 AND #2 AND #3

#5 PY=(2014-2024) AND DT=(Article OR Review)

**CNKI**

#1 SU='高风险妊娠' OR SU='妊娠风险因素' OR TI='高危妊娠' OR AB='妊娠危险因素'

#2 SU='妊娠并发症' OR TI='妊娠不良结局' OR AB='产科并发症'

#3 SU='不良出生结局' OR TI='早产' OR AB='低出生体重' OR SU='死产'

#4 #1 AND #2 AND #3

#5 时间范围：2014-2024年

**SCOPUS**

#1 'high risk pregnancy'/exp OR 'high-risk pregnan*':ti,ab OR 'pregnancy risk factor*':ti,ab

#2 'risk factor'/exp OR 'pregnancy complication'/exp OR 'pregnancy complication*':ti,ab OR 'obstetric complication*':ti,ab

#3 'birth outcome'/exp OR 'low birth weight'/exp OR 'premature labor'/exp OR 'adverse birth outcome*':ti,ab OR 'preterm birth':ti,ab

#4 #1 AND #2 AND #3

#5 [2014-2024]/py AND [humans]/lim AND ('observational study'/exp OR 'cohort analysis'/exp)

#6 #4 AND #5

**MEDLINE (Pubmed)**

#1 "High-Risk Pregnancy"[Mesh] OR "Pregnancy, High-Risk"[Mesh] OR "high risk pregnan*"[Title/Abstract] OR "high-risk pregnan*"[Title/Abstract] OR "risk factor*"[Title/Abstract]

#2 "Risk Factors"[Mesh] OR "Pregnancy Complications"[Mesh] OR "pregnancy complication*"[Title/Abstract] OR "obstetric complication*"[Title/Abstract] OR "adverse pregnancy outcome*"[Title/Abstract]

#3 "Birth Outcomes"[Mesh] OR "Infant, Low Birth Weight"[Mesh] OR "Premature Birth"[Mesh] OR "adverse birth outcome*"[Title/Abstract] OR "neonatal outcome*"[Title/Abstract] OR "preterm birth"[Title/Abstract] OR "stillbirth"[Title/Abstract]

#4 #1 AND #2 AND #3

#5 ("2014/01/01"[Date - Publication] : "2024/12/31"[Date - Publication]) AND "humans"[MeSH Terms] AND "observational study"[Publication Type]

#6 #4 AND #5
